# Minimally invasive interventions for atrophic acne scars: a PubMed/PMC-based systematic evidence review and exploratory network meta-analysis toward phenotype-guided interpretation

**DOI:** 10.3389/fsurg.2026.1943995

**Published:** 2026-09-01

**Authors:** Ya Liu, Zhenguang Zhang, Jing Zhao

**Affiliations:** Department of Burns II, Handan Iron and Steel Hospital, Handan, China

**Keywords:** acne scar, atrophic scar, fractional laser, microneedling, network meta-analysis, platelet-rich plasma

## Abstract

**Background:**

Atrophic acne scars represent heterogeneous structural problems rather than a single treatment indication. This review compared minimally invasive, energy-based, and regenerative interventions and discussed, at a conceptual level, how sparse comparative evidence might generate phenotype-aware hypotheses for future trials.

**Methods:**

This PROSPERO-registered PubMed/PMC-based review (CRD420261471957) searched PubMed/MEDLINE and PMC open-full-text records from inception to 20 June 2026. The database-limited strategy was chosen to prioritize records with verifiable source tables and reproducible aggregate data for exploratory network meta-analysis. Reference lists and accessible trial-registry records were checked for supplementary verification, but Embase, CENTRAL, Web of Science, Scopus, ClinicalTrials.gov, WHO ICTRP, and grey-literature sources were not systematically searched; therefore, eligible studies outside the PubMed/PMC-accessible evidence base may have been missed. Randomized and split-face randomized trials reporting extractable clinical scar-severity outcomes were synthesized using frequentist random-effects network meta-analysis; clinical heterogeneity was assessed by node definitions, treatment protocols, device platforms, outcome scales, and effect modifiers, with sensitivity analyses varying assumed within-person correlations for split-face trials.

**Results:**

Nine randomized trials, 19 treatment arms, 11 direct comparisons, and 10 nodes formed a sparse connected network. Compared with laser monotherapy, numerically lower post-treatment severity point estimates were observed for laser plus PLLA (SMD −0.69, 95% CI −1.55–0.18; 95% prediction interval −1.80–0.42), laser plus PRP (SMD −0.20, 95% CI −0.81–0.40; 95% prediction interval −1.12–0.72), and microneedling plus chemical peel (SMD −0.14, 95% CI −1.34–1.07; 95% prediction interval −1.53–1.25), although all confidence and prediction intervals crossed the null. No closed loop from independent designs was available for formal inconsistency testing. Heterogeneity was moderate to substantial (tau2 = 0.124; I2 = 65.3%, 95% CI 0.0% to 92.1%), most nodes were supported by one or two studies, and sensitivity analyses changed the top-ranked treatment; therefore, *P*-scores were treated only as descriptive exploratory statistics and a reliable treatment ranking does not currently exist. CINeMA rated comparisons as low or very low confidence. No included trial reported treatment effects by scar morphology, so phenotype-based comparative efficacy could not be tested.

**Conclusions:**

Some combination strategies had numerically lower point estimates, but the network is too sparse and uncertain to support a dependable treatment hierarchy or any definitive between-treatment claim. Because included trials did not report phenotype-stratified results, the phenotype-guided component should be read only as a conceptual, hypothesis-generating interpretation and not as phenotype-specific treatment guidance.

**Evidence certainty:**

Low to very low across network comparisons; exploratory therapeutic evidence synthesis rather than a definitive treatment-ranking claim.

**Systematic Review Registration:**

https://www.crd.york.ac.uk/PROSPERO/view/CRD-420261471957, PROSPERO: CRD420261471957.

## 1 Introduction

Atrophic acne scars are common, durable, and psychologically visible sequelae of acne vulgaris (9, 12, 13, 40). Yet the term “acne scar” hides several biologically different problems. Rolling scars reflect dermal tethering and volume loss; boxcar scars combine surface contour change with sharply demarcated edges; ice-pick scars are narrow, deep, and focal; mixed scars contain more than one of these components (7–11). A treatment that remodels texture may not release tethering, and a treatment that fills volume may not correct focal depth. Consequently, a treatment ranking that ignores morphology may be statistically valid but clinically misleading.

This phenotype problem is an important clinical gap, but it was not directly resolved by the available randomized evidence. Procedural options now include fractional lasers, radiofrequency microneedling, mechanical microneedling, chemical peels, PRP, PLLA, hyaluronic acid, subcision, stromal vascular fraction, and other regenerative approaches (14, 18–23, 37–39, 43, 44). They differ in target tissue, mechanism, downtime, cost, and pigmentary risk (14, 38, 41, 42). Nevertheless, many conventional reviews still ask which modality is “best” without first asking which scar phenotype is being treated (14–17).

Previous meta-analyses have therefore been informative but incomplete (14–17). Pairwise syntheses can test whether PRP improves laser or whether microneedling improves a score, but they rarely position multiple procedural families within one comparative framework. Broader reviews often mix response grades, satisfaction scores, ordinal scales, same-class comparisons, and heterogeneous scar morphologies. This creates a transitivity problem before any statistical model is fitted: a rolling-scar trial and an ice-pick-scar trial may not be answering the same clinical question (1, 3, 4). The central question should no longer be only which device is best, but which biological defect requires correction.

Network meta-analysis can organize fragmented randomized evidence, but rankings remain clinically incomplete when scar morphology is not measured or reported (1, 4–6). Rather than identifying a universally superior device, we sought to synthesize the available comparative evidence and discuss how it might inform future phenotype-stratified research questions. The novelty of this review is therefore not a formal phenotype-stratified analysis, but a cautious conceptual reinterpretation of exploratory network evidence in light of scar biology. We synthesized randomized trials with extractable clinical scar-severity outcomes, assessed risk of bias and certainty, and interpreted treatment nodes accordingly (1–3).

## 2 Materials and methods

### 2.1 Protocol and registration

This systematic PubMed/PMC-based evidence review and network meta-analysis was registered in PROSPERO (CRD420261471957). The search strategy was intentionally framed as database-limited because the primary analytic objective required accessible full-text source tables, extractable arm-level summary statistics, and reproducible linkage between bibliographic records and trial reports. Therefore, the review should be interpreted as a systematically conducted but database-limited synthesis rather than an exhaustive all-database systematic review.

### 2.2 Information sources and search strategy

PubMed/MEDLINE and PMC open-full-text records were searched from database inception to 20 June 2026. PubMed/MEDLINE was selected as the primary biomedical bibliographic source, and PMC was used for open-full-text verification because the network meta-analysis required transparent extraction of arm-level means, standard deviations, sample sizes, follow-up time points, and treatment-node details from source tables. Search terms combined acne scar concepts with randomized-trial and intervention terms, including laser, microneedling, radiofrequency, chemical peel, platelet-rich plasma, subcision, filler, PLLA, and stromal vascular fraction. PMIDs, PMCIDs, DOIs, publication types, abstracts, and full-text tables were checked. Reference lists of included studies and relevant reviews were screened, and accessible trial-registry records were checked for supplementary verification. No language restriction was applied at the search stage; however, eligibility for quantitative synthesis required sufficient bibliographic information and extractable aggregate outcome data. Embase, CENTRAL, Web of Science, Scopus, ClinicalTrials.gov, WHO ICTRP, dissertations, conference proceedings, preprint servers, and other grey-literature sources were not systematically searched. This restriction is a major limitation of evidence completeness: the review should not be interpreted as proving that no additional eligible trials exist outside the searched PubMed/PMC-accessible sources. The full reproducible search strategy and database translations are provided in Supplementary Table S1.

The restricted search strategy was chosen for reproducibility and data auditability, not because the omitted databases were considered unimportant. It may have missed randomized trials indexed only in Embase, CENTRAL, Web of Science, or Scopus; unpublished or ongoing trials registered only in ClinicalTrials.gov or WHO ICTRP; non-open-full-text studies; conference abstracts; dissertations; and other grey literature. This limitation directly affects the completeness of the evidence base and may introduce selection, language-accessibility, and publication-status bias. Accordingly, the network geometry, node support, treatment rankings, and phenotype-oriented interpretation should all be read within this restricted evidence-retrieval frame. For this reason, the results are presented as exploratory and hypothesis-generating rather than as a definitive treatment hierarchy.

### 2.3 Study selection

Two reviewers independently screened records by title, abstract, publication type, and full-text eligibility using prespecified criteria. Disagreements or uncertain classifications were resolved by discussion and, when needed, adjudication by a third reviewer. Randomized and split-face randomized trials were considered eligible. Exclusion reasons included active acne without scar-specific outcomes, hypertrophic or keloid scarring, non-randomized design, single-arm design, duplicate cohort, nonclinical endpoints, nonextractable ordinal-only outcomes, and comparisons that did not inform between-node effects. A record-level list of excluded full texts with reasons is provided in Supplementary Table S2.

### 2.4 Eligibility criteria

Eligible studies enrolled patients with facial atrophic acne scars and compared at least two procedural, energy-based, injectable, chemical, or regenerative interventions. Studies were required to report post-treatment scar severity as mean, standard deviation, and sample size, or in a format convertible to these values. The primary analysis used the prespecified medium-term endpoint defined as the longest extractable follow-up between 12 and 24 weeks. When multiple time points were available within this window, the latest time point was selected. Follow-up observations outside the 12–24-week window were not used for the primary network estimate.

### 2.5 Outcome hierarchy

The primary outcome was post-treatment clinical scar severity. ECCA total score and Goodman-Baron quantitative or qualitative scores were prioritized (7, 8). Final post-treatment scores were used because they were the only outcome format that was available with mean, standard deviation, sample size, and treatment-node information across the connected network. Baseline-adjusted or change-from-baseline effects were planned as sensitivity analyses when extractable baseline means, final means, change scores, and change-score standard deviations or sufficient paired information were available. Patient satisfaction, global improvement, device-based skin-analysis metrics, and adverse events were treated as secondary or descriptive outcomes because they represent different clinical constructs.

### 2.6 Intervention nodes and transitivity

Treatment nodes were constructed *a priori* to balance clinical interpretability, biological plausibility, and network connectivity. Laser monotherapy combined fractional CO2 and Er:YAG ablative resurfacing lasers because both were used as resurfacing-laser comparators or anchors for add-on strategies; however, this merge was treated as a transitivity concern rather than as evidence that the two laser systems are interchangeable. Trials also varied in protocol intensity, including number of sessions, treatment interval, laser or RF platform, energy-delivery approach, adjunctive product, and follow-up time; these variables were extracted when reported but could not be modeled because the network was too sparse. The chemical peel node consisted of TCA chemical peeling only, so the abbreviated node label “chemical peel” should be interpreted as TCA peel and not as a pooled estimate across all peel agents. RF microneedling combined NIMFRF and MFR platforms because both deliver fractional radiofrequency energy through microneedles, but platform differences, settings, and adjunctive topical insulin were recorded as potential effect modifiers. Adjunct-specific combination nodes were retained when the adjunct changed the clinical decision context, including laser plus PRP, laser plus PLLA, microneedling plus chemical peel, microneedling plus hyaluronic acid, RF microneedling plus topical insulin, and regenerative/SVF gel. Potential effect modifiers included baseline scar severity, scar morphology, Fitzpatrick skin type, device type, number of sessions, follow-up time, and outcome scale. A structured transitivity assessment and node-fragmentation sensitivity assessment are provided in Supplementary Tables S5 and S9d.

### 2.7 Data extraction

Two reviewers independently extracted study design, population, intervention arms, treatment node, sample size, outcome scale, follow-up time, mean, standard deviation, and source table into a structured extraction form. Device and protocol descriptors, including laser system, RF microneedling platform, adjunctive agent, number of sessions, treatment interval, and selected follow-up window, were extracted when available to support clinical heterogeneity assessment. Baseline means, change-from-baseline scores, and change-score variance data were also sought. Discrepancies were resolved by consensus and adjudicated by a third reviewer when consensus was not reached. One study reported final GSS values as median and interquartile range; for this study, the approximate mean was calculated as (Q1 + median + Q3)/3 and the approximate standard deviation as (Q3 - Q1)/[2 × Phi^{-1}{(0.75n - 0.125)/(*n* + 0.25)}], following Wan et al. (45). This conversion was flagged in the extraction and sensitivity tables because it assumes an approximately symmetric within-arm distribution and may be unstable when applied to a single-study treatment node. Split-face trials were retained because they contributed clinically relevant within-patient randomized evidence, but paired mean differences, paired standard errors, or within-person correlations were not consistently reported. The primary analysis therefore used aggregate arm-level final scores. A pure within-treatment comparison model was not used because it would require paired contrast estimates and within-person covariance information for each split-face comparison, which were not available across the network.

### 2.8 Risk of bias and certainty assessment

Risk of bias was assessed using RoB 2 domains: randomization process, deviations from intended interventions, missing outcome data, outcome measurement, and selection of reported result. Confidence in network estimates was summarized using CINeMA domains: within-study bias, reporting bias, indirectness, imprecision, heterogeneity, and incoherence (3). For the CINeMA imprecision domain, no validated minimally important difference (MID) was available that could be applied consistently across ECCA, Goodman-Baron, and Goodman global scarring scales. Therefore, before final certainty grading, the review team prespecified an SMD of 0.50 as a conservative cross-scale MID, corresponding to a moderate standardized clinical difference. Because lower scar-severity scores indicate better outcomes, SMD <= −0.50 was considered a clinically important benefit and SMD >= 0.50 a clinically important disadvantage. Major imprecision concerns were assigned when the 95% CI crossed the null and included at least one MID boundary; the rule and comparison-level judgments are shown in Supplementary Table S7.

### 2.9 Statistical analysis

Continuous outcomes were synthesized as standardized mean differences, with lower scar-severity scores representing better outcomes. A frequentist random-effects network meta-analysis was conducted using R 4.5.3, netmeta 3.6–0, and meta 8.5–0. Treatment ranking was summarized with *P*-scores only as a descriptive exploratory statistic because the network contained few trials, several single-study nodes, and no independent-design closed loop for formal inconsistency testing (4–6). Heterogeneity was quantified using tau2, tau, and I2, and 95% prediction intervals were calculated from the treatment-specific standard error and the common between-study standard deviation (tau). Because ECCA, Goodman-Baron quantitative/qualitative scales, and GSS-type outcomes differ in construct and scaling, SMDs were used only to place continuous scar-severity outcomes on a common metric; this standardization does not remove clinical or measurement heterogeneity. The number of studies and arm-level sample size contributing to each node were tabulated, and the contribution structure was summarized in Supplementary Figure S3 and Supplementary Table S10. The Rybak three-arm trial created a within-study triangle among chemical peel, microneedling, and microneedling plus chemical peel, but no closed loop from independent designs was available for inconsistency testing.

### 2.10 Sensitivity, safety, and small-study effects

Sensitivity analyses examined exclusion of the median/IQR-converted PLLA study, exclusion of high overall risk-of-bias studies, restriction to nodes with direct laser comparison, the influence of split-face variance assumptions, and the feasibility of splitting clinically heterogeneous nodes. The median/IQR conversion sensitivity was prespecified as an influence check because the converted study was the only source for the laser plus PLLA node and therefore could affect both the point estimate and the exploratory ranking. For split-face studies, approximate contrast variances were recalculated using assumed within-person correlations of 0, 0.25, 0.50, and 0.75 (Supplementary Table S9b). Feasibility of baseline-adjusted and change-from-baseline sensitivity analysis was assessed at the study level. A formal change-score or baseline-adjusted NMA was not estimable because baseline values and change-score variances were not consistently extractable across connected treatment nodes (Supplementary Table S9c). For node-level sensitivity, we attempted to split the laser node by CO2 versus Er:YAG and the RF microneedling node by NIMFRF versus MFR platform; the resulting network connectivity and interpretability were assessed before model estimation (Supplementary Table S9d). Safety outcomes were extracted into a structured table by study and intervention, recording event counts when reported, severity definitions, time points or follow-up windows, infection/scarring outcomes, serious adverse events, and whether an item was not reported or only qualitatively described.

## 3 Results

### 3.1 Study selection

**Figure 1:**
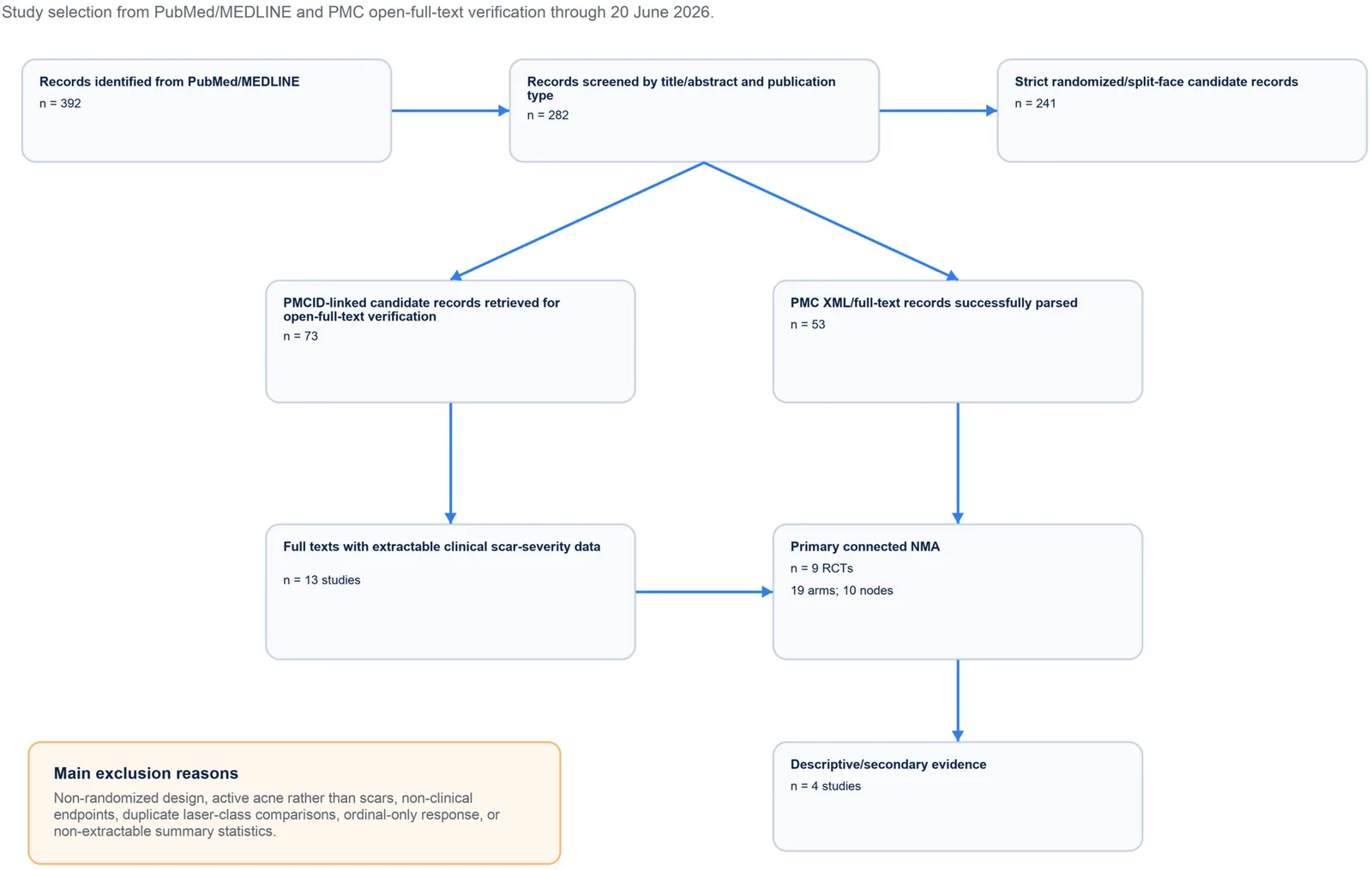
PRISMA-style flow diagram for study selection and evidence assembly.

The PubMed/MEDLINE inventory identified 392 records. After deduplication and title/abstract/publication-type screening, 282 records remained, and 241 met strict randomized or split-face candidate criteria. Seventy-three PMCID-linked records were retrieved for full-text verification; 53 PMC XML/full-text records were successfully parsed for detailed eligibility and extractability assessment. Records were excluded at the full-text stage mainly because they were not randomized or split-face randomized trials, did not enroll patients with facial atrophic acne scars, did not report an extractable continuous scar-severity outcome, duplicated another cohort, or tested a comparison that could not inform the connected treatment network. Thirteen studies had extractable clinical scar-severity or close contextual evidence: 9 entered the primary connected NMA and 4 were retained only for descriptive/contextual interpretation, with reasons listed in Supplementary Table S13 (Figure 1).

### 3.2 Study characteristics and network geometry

The primary network included 9 randomized trials, 19 treatment arms, 11 direct comparisons, and 10 treatment nodes. Five trials used split-face designs and four used parallel-group designs. All primary network estimates used follow-up points within the prespecified 12–24-week window; selected endpoints ranged from 12 to 24 weeks. Because the split-face reports did not consistently provide paired mean differences, paired standard errors, or within-person correlations, their contribution to precision was evaluated in assumed-correlation sensitivity analyses.

**Figure 2:**
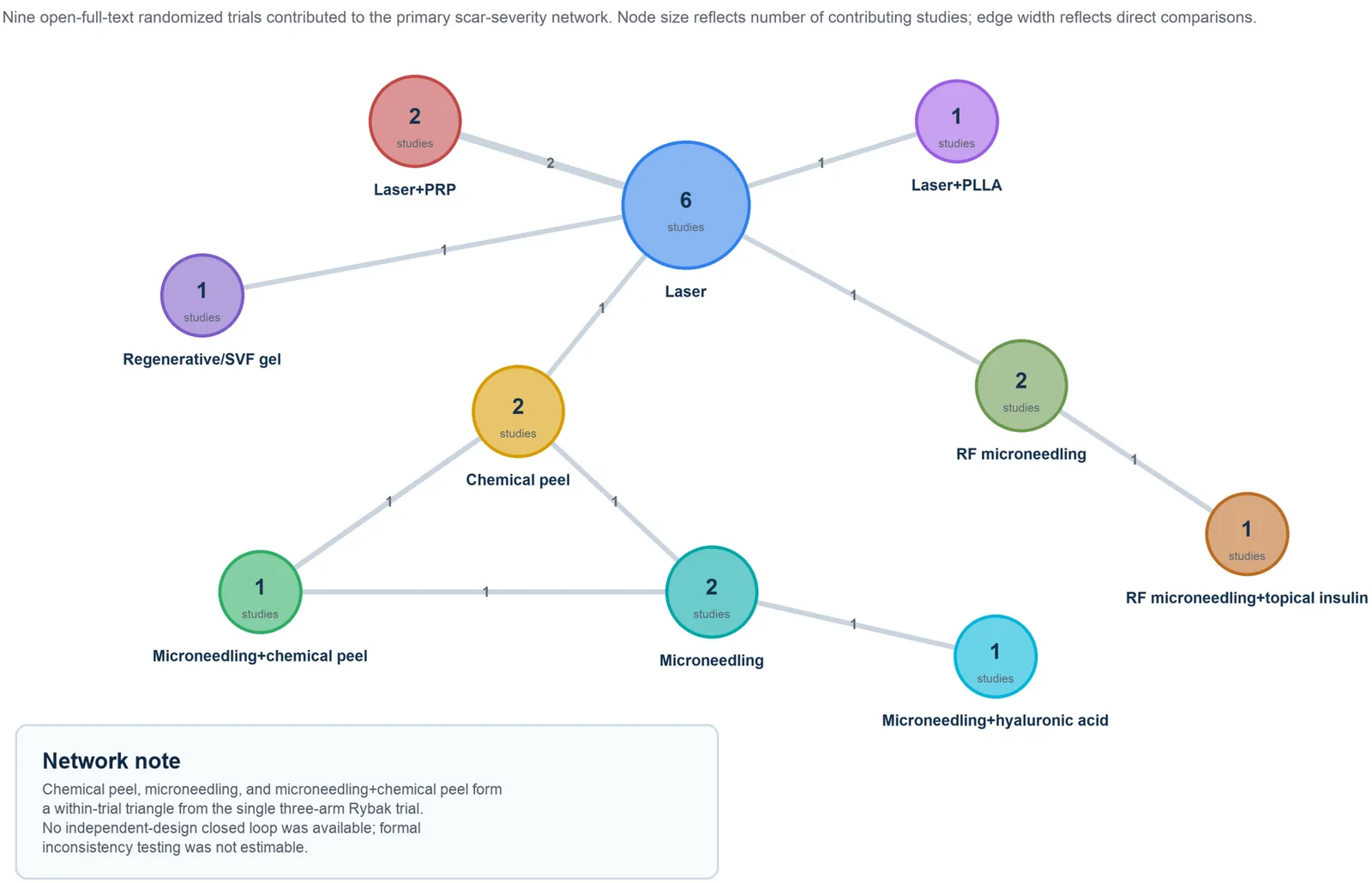
Primary connected treatment-class network. The chemical peel, microneedling, and microneedling plus chemical peel triangle comes from the single three-arm Rybak trial and does not provide independent-design evidence for formal inconsistency testing.

Node-level evidence was limited: the reference laser node included six studies and 157 arm-level observations; chemical peel, laser plus PRP, microneedling, and RF microneedling each included two studies; all other active nodes were supported by a single study with 10–38 arm-level observations (Supplementary Table S12) (Table 1, Figure 2).

**Table 1:** Study characteristics.

Study	PMID	PMCID	Design	Arm n	Treatment nodes	Outcome	Weeks
Galal et al. (29) FCO2 PRP	37266087	PMC10231723	split-face	64	Laser + PRP; Laser	Goodman-Baron quantitative score	18
Shah et al. (28) FACL PRP	34084007	PMC8149982	split-face	60	Laser + PRP; Laser	Goodman-Baron qualitative scar grade	16
Khurana et al. (31) ErYAG TCA	38314354	PMC10833483	parallel	50	Laser; Chemical peel	Goodman-Baron quantitative score	12
Zhang et al. (35) SVF CO2	40112029	PMC11925327	split-face	20	Regenerative/SVF gel; Laser	ECCA total score	24
Rybak-D'Obyrn et al. (30) MN CP	34658706	PMC8501429	parallel	119	Microneedling + chemical peel; Chemical peel; Microneedling	Goodman-Baron scale	12
Bano et al. (32) MN HA	38314374	PMC10833484	parallel	60	Microneedling; Microneedling + hyaluronic acid	Goodman quantitative global scarring grade system	17
Wu et al. (33) NIMFRF CO2	40568948	PMC12228042	split-face	60	RF microneedling; Laser	ECCA total score	24
Sirithanabadeekul et al. (34) MFR TI	39970082	PMC11838817	split-face	58	RF microneedling + topical insulin; RF microneedling	ECCA total score	24
Zhou et al. (36) PLLA CO2	40476635	PMC12143118	parallel	60	Laser; Laser + PLLA	Goodman quantitative global scarring grade system	24

### 3.3 Transitivity and clinical heterogeneity

Important effect modifiers were incompletely reported across studies. Trials differed in device class, laser type (CO2 versus Er:YAG), RF microneedling platform, adjunctive biologic or injectable use, baseline scar severity, skin phototype, number of sessions, treatment interval, follow-up, and outcome scale. The analyzable laser evidence included both CO2-centered and Er:YAG-centered comparisons, but these lasers may differ in ablation depth, thermal injury profile, downtime, and pigmentary risk; therefore, the pooled laser node should be interpreted as a resurfacing-laser anchor rather than as evidence for a specific laser system. The chemical peel evidence in the analyzable network was limited to TCA peel. No included trial reported treatment effects stratified by rolling, boxcar, ice-pick, or mixed scar morphology, and scar-phenotype distributions were absent or insufficiently detailed for matched phenotype-intervention analysis. Asian populations and Fitzpatrick III-IV skin types were prominent in several recent studies, making pigmentary safety clinically relevant.

### 3.4 Risk of bias

**Figure 3:**
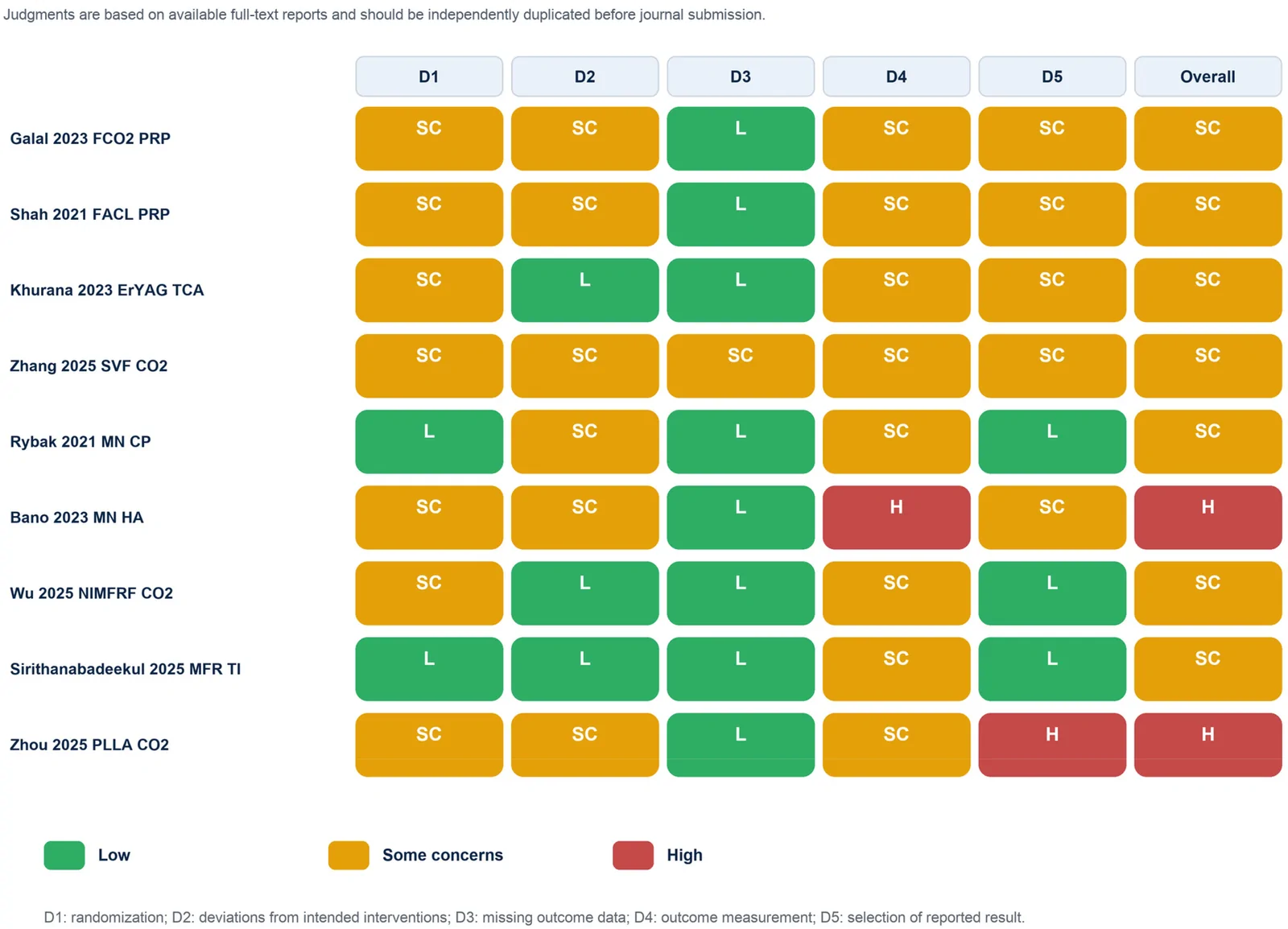
Risk-of-bias assessment using RoB 2 domains.

Two trials were judged at high overall risk of bias. The main concerns were open-label or subjective outcome measurement, incomplete reporting of allocation concealment or assessor masking, selective-reporting concerns, and reliance on converted summary statistics. The remaining trials were judged as having some concerns (Figure 3).

### 3.5 Primary network meta-analysis

**Figure 4:**
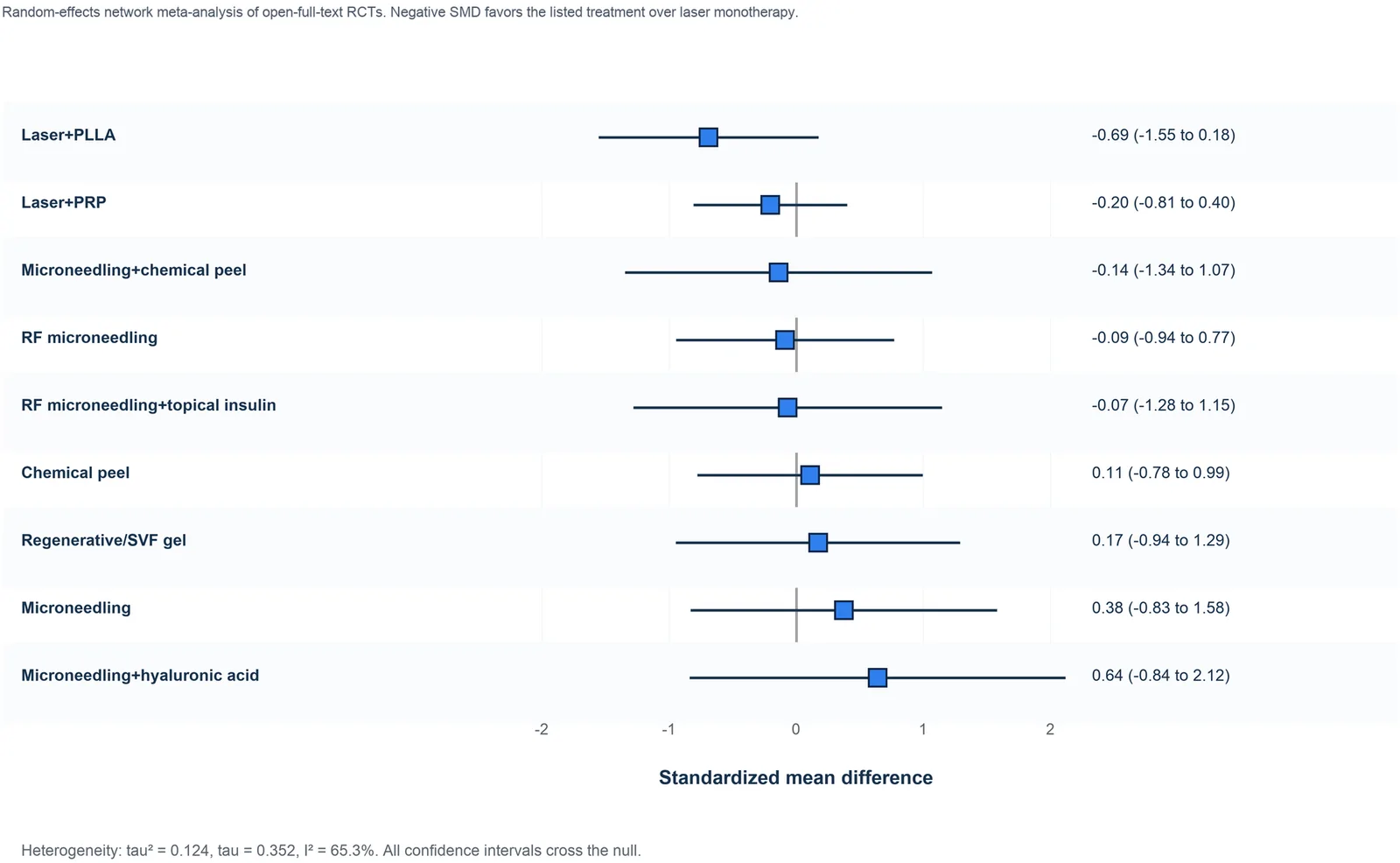
Random-effects network estimates versus laser monotherapy.

No comparison showed statistically conclusive superiority over laser monotherapy. Laser plus PLLA had the largest favorable point estimate and highest *P*-score, but this node was supported by a single study with converted summary statistics and its confidence and prediction intervals crossed the null. Across all active comparisons versus laser, 95% prediction intervals were wider than confidence intervals and included the null, indicating that the expected effect in a future comparable study remains uncertain. Because most nodes were supported by one or two studies, there was no independent-design loop for formal inconsistency testing, and rankings changed after excluding the PLLA study or high-risk-of-bias studies, the *P*-score order should not be interpreted as a clinically reliable hierarchy (Table 2, Figure 4).

**Table 2:** Network meta-analysis estimates versus laser monotherapy.

Treatment vs. laser	SMD	95% CI	95% prediction interval	*P*-score	Confidence
Laser plus PLLA	−0.69	−1.55–0.18	−1.80–0.42	0.865	Very low
Laser plus PRP	−0.20	−0.81–0.40	−1.12–0.72	0.635	Low
Microneedling plus chemical peel	−0.14	−1.34–1.07	−1.53–1.25	0.611	Very low
RF microneedling	−0.09	−0.94–0.77	−1.19–1.01	0.549	Low
RF microneedling plus topical insulin	−0.07	−1.28–1.15	−1.47–1.33	0.531	Very low
Chemical peel	0.11	−0.78–0.99	−1.01–1.23	0.438	Low
Regenerative/SVF gel	0.17	−0.94–1.29	−1.14–1.48	0.404	Very low
Microneedling	0.38	−0.83–1.58	−1.01–1.77	0.292	Very low
Microneedling plus hyaluronic acid	0.64	−0.84–2.12	−0.99–2.27	0.191	Very low

### 3.6 Heterogeneity and inconsistency

Between-study heterogeneity was moderate to substantial (tau2 = 0.124; tau = 0.352; I2 = 65.3%, 95% CI 0.0% to 92.1%). This statistical heterogeneity is clinically plausible because included trials differed in laser systems, RF platforms, treatment protocols, adjunctive products, scar-severity scales, follow-up time, and population phototype. Prediction intervals for all comparisons versus laser crossed the null, reinforcing that the observed favorable point estimates should not be interpreted as stable superiority claims. A triangular structure was present among chemical peel, microneedling, and microneedling plus chemical peel, but all three edges came from the same three-arm Rybak trial. Therefore, no closed loop from independent designs was available, and local inconsistency and design-by-treatment inconsistency models were not estimable. This geometry limits the ability of the network to detect disagreement between direct and indirect evidence and further reduces confidence in any treatment ordering.

### 3.7 Certainty of evidence

**Figure 5:**
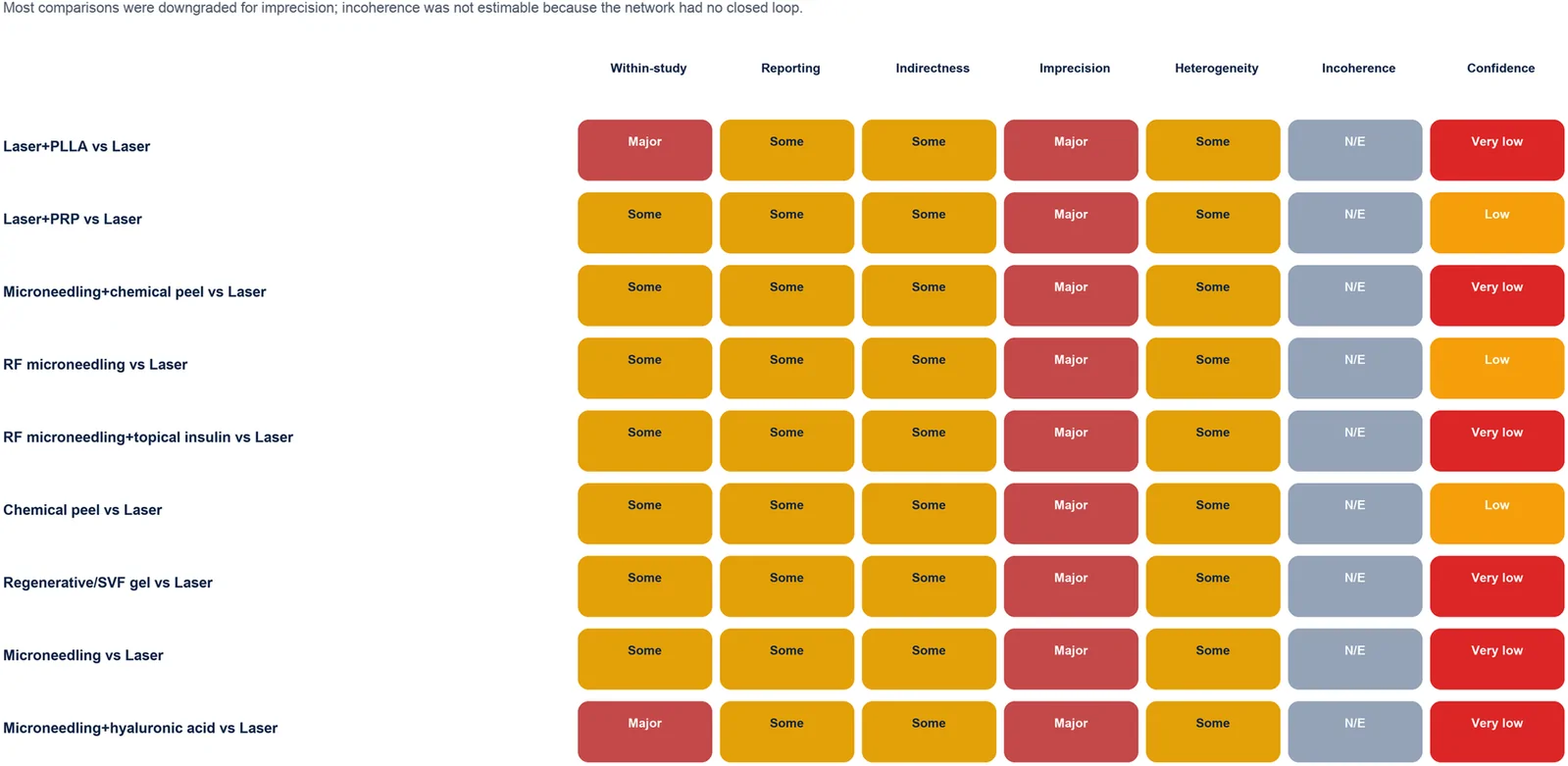
CINeMA-domain confidence assessment.

CINeMA-domain assessment rated most comparisons as low or very low confidence. All comparisons had major imprecision concerns because their 95% CIs crossed the null and included at least one prespecified MID boundary (SMD <= −0.50 for clinically important benefit or SMD >= 0.50 for clinically important disadvantage). Downgrading was also driven by sparse single-study nodes, within-study bias, moderate-to-substantial heterogeneity, and inability to assess incoherence (Figure 5).

### 3.8 Safety synthesis

Safety outcomes were reported heterogeneously and could not be pooled. Structured extraction showed that event counts were absent or only qualitative in several trials; therefore, qualitative reports were not treated as quantitative safety evidence. The most complete comparative safety data came from the NIMFRF versus AFCO2 split-face trial: pain occurred in all participants on both sides but was lower with NIMFRF (median VAS 3 vs. 8), PIH occurred in 2/30 versus 11/30 sides, and infections, blisters, erosions, hypopigmentation, acneiform eruption, and worsening scarring were not observed on either side. The ECM/SVF gel versus CO2 laser study reported no CTCAE-defined adverse events, no infections or secondary scarring, and mild localized pigmentation in 2/10 CO2-treated sides. The CO2 laser plus PLLA trial reported moderate pain (NRS 4–7), erythema in 4/30 laser-alone patients, PIH in 2/30 laser-alone patients, erythema/bruising in 10/30 combination patients, and no nodules, hypertrophic scars, or severe adverse reactions. Across the remaining studies, pain, erythema, edema, crusting/scabbing, PIH, bruising, or acneiform eruption were generally transient but were often reported without denominators, severity grades, or standardized follow-up windows (Supplementary Table S8).

### 3.9 Sensitivity and contribution structure

Excluding the median/IQR-converted PLLA study shifted the top-ranked treatment from laser plus PLLA to laser plus PRP, showing that the highest *P*-score depended on a single converted-summary-statistic study. The favorable direction of combination strategies was otherwise not reversed, but the exclusion made laser plus PLLA non-estimable and reinforced that rankings should not be treated as reliable comparative evidence. Excluding high overall risk-of-bias studies produced the same directional shift. In split-face sensitivity analyses assuming within-person correlations of 0, 0.25, 0.50, and 0.75, the direction of the principal estimates and the absence of statistically conclusive superiority were unchanged; laser plus PLLA remained the most favorable point estimate because it came from a parallel-group study, whereas confidence intervals for split-face-dependent nodes varied with the assumed correlation (Supplementary Table S9b). Baseline-adjusted and change-from-baseline NMA could not be estimated because baseline means and change-score standard deviations or paired covariance information were not consistently extractable across the connected network (Supplementary Table S9c). Node-fragmentation sensitivity showed that splitting laser monotherapy into CO2 and Er:YAG components separated the CO2-centered comparisons from the Er:YAG/TCA/microneedling component, and splitting RF microneedling into NIMFRF and MFR platforms disconnected the RF microneedling plus topical-insulin estimate. These findings support cautious, mechanism-based interpretation rather than a definitive rank order.

## 4 Discussion

This network meta-analysis should be interpreted as an exploratory evidence map rather than as a rank-defining comparative effectiveness analysis. Combination procedural strategies had directionally favorable point estimates, but the sparse network, single-study nodes, wide confidence and prediction intervals, moderate-to-substantial heterogeneity, absence of independent-design inconsistency evidence, and ranking changes under sensitivity analyses mean that a reliable treatment ranking does not currently exist. The useful signal is therefore hypothesis-generating: multimodal approaches that combine resurfacing with regenerative, volumizing, or remodeling components may deserve further testing in phenotype-stratified trials.

This distinction matters because acne scars are structurally heterogeneous (7–11). Rolling scars often reflect dermal tethering and volume loss, boxcar scars have sharper contour defects, and ice-pick scars are focal, deep tracts. However, these morphology categories were not available as analyzable subgroups in the included trials. The following phenotype discussion is therefore a conceptual interpretation for future study design, not evidence that any intervention has proven phenotype-specific superiority.

### 4.1 Toward phenotype-guided interpretation

This review challenges the traditional assumption that acne scar procedures should be ranked primarily by device category, but it does not validate a phenotype-guided treatment algorithm. The familiar clinical pathway is device-forward: a patient presents with acne scars, and the decision becomes laser, PRP, microneedling, RF, or filler (14–17). A phenotype-aware research perspective would reverse that sequence by considering morphology, skin phototype, biological defect, downtime tolerance, and patient priority before testing a procedural strategy (7–11, 41, 42).

In this conceptual framework, treatment selection is presented only as biological reasoning to be tested. Tethering calls for release, volume loss for support or collagen stimulation, surface irregularity for resurfacing, focal depth for focal reconstruction, and pigmentary vulnerability for epidermal-sparing choices. Because no included study stratified outcomes by rolling, boxcar, ice-pick, or mixed morphology, this framework should be regarded as hypothesis-generating clinical reasoning rather than evidence of superiority for phenotype-based management.

### 4.2 Mechanistic interpretation

The favorable direction for combination therapy is biologically plausible and consistent with established scar-remodeling concepts. Fractional lasers and RF microneedling induce controlled dermal injury and collagen remodeling; PRP has been studied as a platelet-derived growth-factor source for wound repair; PLLA has been used as a collagen-stimulating volumizing agent; peels and microneedling target superficial texture, dyspigmentation, and percutaneous remodeling (18–23, 37–39, 43, 44). These mechanisms are supported by prior acne-scar, wound-healing, and procedural-dermatology literature, but the present network cannot prove which component drove benefit.

The apparent advantage of laser plus PLLA should therefore be read as a mechanistically plausible signal, not a practice-changing result (35, 36). Laser plus PRP is similarly attractive because biologic support after controlled injury may aid repair, but its estimate remained small and imprecise (15, 22–29). Mechanical microneedling and RF microneedling also require nuance: the former may improve texture and collagen induction, whereas the latter may be useful in pigment-prone patients because dermal thermal injury can be delivered with less epidermal disruption than fully ablative resurfacing (20, 21, 33, 34, 38, 41–43).

Rolling scars deserve special emphasis. Their dominant pathology is often fibrous septal tethering, sometimes combined with dermal or subcutaneous volume loss (7–11). Resurfacing alone may improve overlying texture but cannot reliably release a tether. This is why subcision, with or without volumizing or collagen-stimulating adjuncts, remains mechanistically central for rolling scars even when it is underrepresented in the analyzable network (9, 37).

Boxcar scars pose a different problem. Shallow boxcar scars may respond to resurfacing because their main defect is texture and edge blending (18, 19, 38). Deeper sharply bordered boxcar scars may require a release or focal reconstruction component before resurfacing can meaningfully improve contour. Ice-pick scars are different again: their narrow epithelialized tracts explain why focal chemical reconstruction such as TCA CROSS is biologically rational, even though many TCA CROSS studies report ordinal outcomes or comparisons that could not be harmonized into the primary continuous severity network (9, 30, 31). Their limited representation should not be interpreted as clinical irrelevance. It reflects a measurement and reporting problem.

### 4.3 Conceptual framework for future phenotype-stratified research

**Figure 6:**
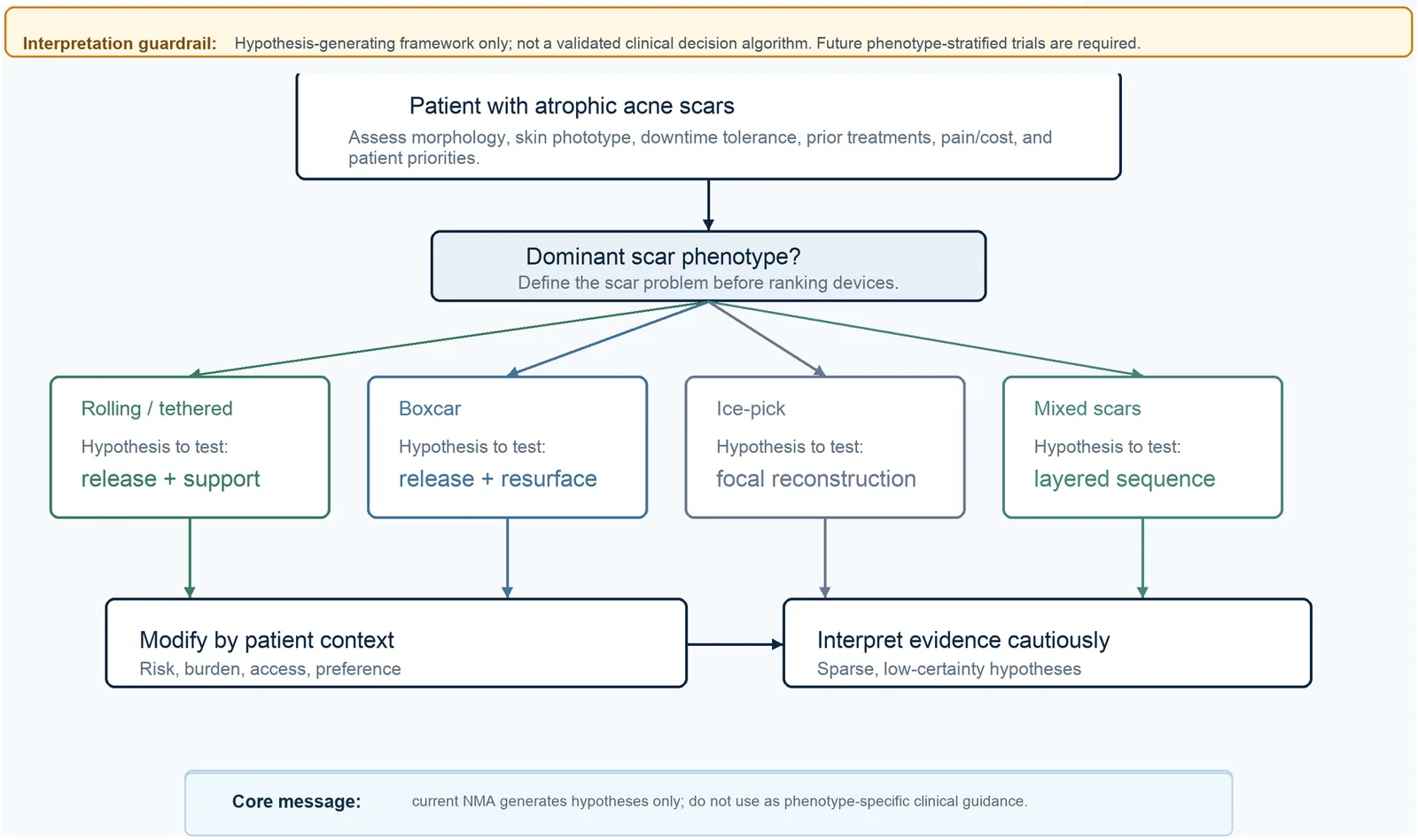
Hypothesis-generating conceptual framework toward phenotype-guided interpretation of atrophic acne scar treatment. The schematic illustrates how scar morphology, pigmentary risk, downtime tolerance, and evidence certainty could generate future research hypotheses rather than relying on modality ranking alone; it was not derived from phenotype-stratified comparative evidence and should not be used as a validated clinical decision algorithm or phenotype-specific treatment guidance.

A practical future research framework should begin with scar phenotype rather than device availability. Rolling scars may require tether release and volume support; boxcar scars often require resurfacing, sometimes with release; ice-pick scars may require focal reconstruction; mixed scars usually need staged combination therapy. Skin phototype, pigmentary risk, downtime tolerance, cost, pain, repeat-session burden, access to trained operators, and patient preference should modify future trial design and shared clinical discussion. The schematic is intended as a conceptual map for hypothesis generation, not as a validated treatment algorithm (Figure 6).

### 4.4 Multimodal reconstruction and precision procedural dermatology

The favorable point estimates for some combination nodes should not be reduced to the idea that adding a second product is automatically better. The more important concept is multimodal reconstruction: mechanical release for tethering, biologic stimulation or volumizing support for dermal and subdermal deficiency, and surface remodeling for texture, edge blending, and dyschromia. Acne scar management may move toward precision procedural dermatology, but the present evidence remains insufficient to validate phenotype-specific treatment rules prospectively.

### 4.5 Relation to previous reviews

Previous acne scar reviews and NMAs have synthesized broader treatment categories, response grades, satisfaction outcomes, and photoelectric modalities (14–17). Those reviews are valuable, but many rank procedures as interchangeable candidates in a league table. Existing guidance acknowledges scar morphology, but comparative evidence has rarely been able to test morphology-specific effects (7–11). Put simply, previous NMAs ranked procedures; the present NMA shows why such rankings remain unreliable in the current evidence network and uses the sparse comparative data only to outline a phenotype-aware research agenda.

This narrower approach reduces network size and statistical power, but it makes the clinical question cleaner. A network that ranks devices without acknowledging rolling, boxcar, ice-pick, and mixed scars risks producing a technically precise but clinically blunt answer.

### 4.6 Methodological considerations

Clinical heterogeneity is central to interpretation. The laser node pooled CO2 and Er:YAG resurfacing lasers, the RF microneedling node pooled different device platforms, and the chemical peel node was in practice a TCA-only node. Within these broad categories, studies varied in treatment intensity, number of sessions, treatment intervals, adjunctive PRP, PLLA, hyaluronic acid, topical insulin, or SVF/ECM gel use, follow-up timing, and post-treatment care. Outcome measures also differed: ECCA, Goodman-Baron quantitative or qualitative scales, and GSS-type scores do not necessarily weight depth, texture, edge definition, and patient-visible improvement in the same way. We retained broad nodes to keep a connected network and to reflect clinically recognizable decision categories, but the node-fragmentation sensitivity analysis showed that more granular device-level splitting would disconnect the evidence rather than produce a stable alternative ranking. Therefore, the present estimates should be read as exploratory category-level comparisons, not as device-specific evidence for a particular wavelength, RF platform, energy setting, needle depth, session schedule, or peel formulation.

Split-face designs have complementary strengths and statistical liabilities. Within-person randomization reduces confounding by age, baseline scar biology, skin phototype, wound-healing tendency, and acne history, which is valuable in procedural dermatology. However, the statistical unit is the paired within-person contrast, not two independent facial sides. If paired mean differences, paired standard errors, or within-person correlations are unavailable, treating split-face arms as independent aggregate arms can overstate the effective amount of independent information and can misestimate precision and weights inside a sparse network. The exact standard-error distortion depends on the unreported within-person covariance and on whether an arm-level or contrast-level approximation is used. Assumed-correlation sensitivity analyses did not change the qualitative conclusions, but they cannot replace trial-reported paired contrast statistics or individual participant data. The absence of closed loops from independent designs prevented formal inconsistency testing, making biological plausibility, transitivity assessment, and certainty grading especially important. *P*-scores were therefore retained only to describe the observed model ordering and should not be used as a treatment algorithm.

### 4.7 Future research

Future acne scar trials should be designed around scar phenotype and should use standardized safety reporting. Trials should report rolling, boxcar, ice-pick, and mixed-scar proportions; Fitzpatrick skin type; baseline severity; prior isotretinoin or procedural treatment; device parameters; number of sessions; treatment intervals; anesthesia; post-procedure care; downtime; pain; pigmentary events; and patient satisfaction. Split-face trials should report paired mean differences, paired standard errors or confidence intervals, within-person correlations, and side-level adverse-event denominators; parallel trials should report baseline-adjusted and change-score data. Safety tables should provide denominators, event definitions, severity grades, onset, duration, resolution, infection, scarring, nodule/granuloma formation for injectables, hypoglycemic symptoms for topical insulin, and serious adverse events. Outcomes should include validated continuous scar-severity scales, standardized photography, blinded assessment, and clinically meaningful responder thresholds. Until such data exist, phenotype-guided interpretation should remain conceptual and hypothesis-generating.

Most importantly, future comparative trials should evaluate staged or combination strategies that reflect real practice. A trial of laser alone versus PRP alone may be less informative than a phenotype-stratified trial asking whether adding a biologic, volumizing, release, or resurfacing component improves the scar component it is supposed to target. Individual participant data network meta-analysis may ultimately test phenotype-specific treatment selection, but the current aggregate literature does not yet support that claim.

### 4.8 Limitations

Several limitations should temper interpretation. First and most importantly for evidence retrieval, although the search and screening process was systematic within PubMed/MEDLINE and PMC, Embase, CENTRAL, Web of Science, Scopus, ClinicalTrials.gov, WHO ICTRP, and grey-literature sources were not systematically searched. The PubMed/PMC focus improved traceability of source tables and reproducibility of arm-level extraction, but it also means that non-indexed, unpublished, non-open-full-text, database-specific, and registry-only trials may have been missed. Therefore, the completeness of the evidence base is limited, and the present network should be interpreted as a transparent PubMed/PMC-accessible evidence synthesis rather than a complete map of all eligible trials. The direction of this potential bias is uncertain: omitted small or negative studies could attenuate apparent benefits, whereas omitted positive studies could strengthen some comparisons. Second, the network was sparse, with several single-study nodes. Third, clinical heterogeneity was substantial: laser systems, RF platforms, peel formulation, adjunctive agents, treatment schedules, follow-up windows, scar-severity scales, and population phototype differed across trials, and these effect modifiers could not be adjusted in meta-regression. Fourth, five studies used split-face designs, but paired contrast statistics were incompletely reported; assumed-correlation sensitivity analyses were therefore exploratory and cannot fully correct for missing paired information. Fifth, no included study reported treatment effects stratified by rolling, boxcar, ice-pick, or mixed scar morphology, so no matched phenotype-intervention analysis was possible and the phenotype-guided component remains conceptual rather than validated. Sixth, the primary endpoint used final post-treatment scores at the longest extractable 12–24-week follow-up; this choice may be affected by baseline imbalance and scale differences, and a formal baseline-adjusted or change-from-baseline NMA was not possible because change-score variances and paired covariance information were incomplete. Seventh, one laser plus PLLA study required conversion from median/IQR to approximate mean/SD using distributional assumptions; because this study defined a single-study node, its conversion could influence the apparent ranking, as confirmed by the exclusion sensitivity analysis. Finally, adverse events were reported heterogeneously and often qualitatively, limiting quantitative safety comparison.

## 5 Conclusion

Laser plus PLLA, laser plus PRP, and microneedling plus chemical peel had numerically lower point estimates, but all confidence and prediction intervals were wide, certainty was low or very low, the network contained several single-study nodes, no independent-design closed loop was available for inconsistency testing, and sensitivity analyses changed the top-ranked treatment. Clinical heterogeneity across device systems, protocols, outcome measures, and split-face reporting further limits any device-specific inference. A reliable treatment ranking does not currently exist, and the present network does not provide phenotype-specific comparative efficacy or a basis for choosing phenotype-specific treatments. This work should instead be viewed as an exploratory NMA accompanied by a conceptual, hypothesis-generating framework toward phenotype-guided interpretation. The next evidence step is to test scar-specific strategies and to ask which biologic problem each procedure solves.

## Data Availability

The original contributions presented in the study are included in the article/Supplementary Material, further inquiries can be directed to the corresponding author.
