# Supplementary material for: Minimally invasive interventions for atrophic acne scars: a PubMed/PMC-based systematic evidence review and exploratory network meta-analysis toward phenotype-guided interpretation
Source: Front Surg. 2026 Sep 1;13:1943995. doi: 10.3389/fsurg.2026.1943995 (PMC13574651; doi:10.3389/fsurg.2026.1943995)
Supplement: Supplementary file 1 [file Table1.docx]

\# Exploratory frequentist network meta-analysis for atrophic acne scar manuscript

\# Requires: R >= 4.5.3, netmeta >= 3.6-0, meta >= 8.5-0

library(netmeta)

library(meta)

dat <- read.csv("Supplementary_Data_Primary_NMA_R1_all_R2_issue4.csv", stringsAsFactors = FALSE)

dat$n <- as.numeric(dat$n)

dat$mean <- as.numeric(dat$mean)

dat$sd <- as.numeric(dat$sd)

pw <- pairwise(

treat = treatment_node,

mean = mean,

sd = sd,

n = n,

studlab = study_id,

data = dat,

sm = "SMD"

)

nma <- netmeta(

TE = pw$TE,

seTE = pw$seTE,

treat1 = pw$treat1,

treat2 = pw$treat2,

studlab = pw$studlab,

sm = "SMD",

common = FALSE,

random = TRUE,

reference.group = "Laser",

details.chkmultiarm = TRUE

)

print(summary(nma))

print(netrank(nma, small.values = "good"))

forest(nma, ref = "Laser", sortvar = TE.random)

netgraph(nma)

\# Sensitivity analyses reported in the manuscript were run by:

\# 1. Removing Zhou_2025_PLLA_CO2 for median/IQR-conversion sensitivity.

\# 2. Removing studies judged high overall RoB.

\# 3. Recalculating split-face contrast variances under assumed correlations.

\# 4. Attempting device-level node fragmentation; formal NMA was not estimable after network disconnection.
